# Supplementary material for: Heat adaptation of phage T7 under an extended genetic code
Source: Virus Evol. 2021 Dec 1;7(2):veab100. doi: 10.1093/ve/veab100 (PMC8923235; doi:10.1093/ve/veab100)
Supplement: veab100_Supp [file veab100_supp.zip › Sequencing_Analysis.zip!/Sequencing_Analysis/S3/evidence/JC_SIDE_1_44.html]

BRESEQ :: Evidence


| New junction evidence | | | | | | | | | | | |
| --- | --- | --- | --- | --- | --- | --- | --- | --- | --- | --- | --- |
|  | | seq id | position | reads (cov) | reads (cov) | score | skew | freq | annotation | gene | product |
| \* | ? | NC\_001604.1 | = 281 | 1619 (1.060) | 375 (0.230) +TAAAGACCAGACC | 16/508 | 34.0 | 17.7% | intergenic (–/–) | *–/–* | –/– |
|  | ? | NC\_001604.1 | 282 = | 1822 (1.060) |  |  |  |  | intergenic (–/–) | *–/–* | –/– |
| Rejected: Coverage evenness skew score above cutoff. | | | | | | | | | | | |
| Rejected: Frequency below/above cutoff threshold. | | | | | | | | | | | |

Only 100 of 2200 total aligned reads displayed.  

`‑‑TCTCACAGTGTACGGACCTAAAGTTCCCCCATA‑GGGGGTACCTAAAGCCCAGCCAATCACCTAAAGTCAACCTTCGGTTGACCTTGAGGGTTCCCTAAGGGTTGGGGATGACCCTTGGGTTTGTCTTTGGGTGTTACCTTGAGTGTCTCTCTGTGTCCCTATCTGTTACAGTCTCCTAAAGTATCCTCCTAAAGTCACCTCCTAACGTCCATCCTAAAGCCAACACCTAAAGCCTACACCTAAAGACCCATCAAGTCAACGCCTATCTTAAAGTTTAAACATAAAGACCAGACCTAAAGACCAGACCTAAAGACACTACATAAAGACCAGACCTAAAGACGCCTTGTTGTTAGCCATAAAGTGATAACCTTTAATCATTGTCTTTATTAATACAACTCACTATAAGGAGAGACAACTTAAAGAGACTTAAAAGATTAATTTAAAATTTATCAAAAAGAGTATTGACTTAAAGTCTAACCTATAGGATACTTACAGCCATCGAGAGGGACACGGCGAATAGCCATCCCAATCGACACCGGGGTCAACCGGATAAGTAGACAGCCTGATAAGTCGCACGAAAAA  >  NC_001604.1/1‑582`  

                                                                                                                                                                                                                                                                                           `|`                                                                                                                                                                                                                                                                                                               
`aagagacagggtaaCGGACCTAAAGTTCCCCCATA‑GGGGGTACCTAAAGCCCAGCCAATCACCTAAAGTCAACCTTCGGTTGACCTTGAGGGTTCCCTAAGGGTTGGGGATGACCCTTGGGTTTGTCTTTGGGTGTTACCTTGAGTGTCTCTCTGTGTCCCTATCTGTTACAGTCTCCTAAAGTATCCTCCTAAAGTCACCTCCTAACGTCCATCCTAAAGCCAACACCTAAAGCCTACACCTAAAGACCCATCAAGTCAACGCCTATCTTAAAGTTTAAACAtaaagaccagacctaaag                                                                                                                                                                                                                                                                                             <  1:71785/288‑1 (MQ=255)`  
         `gtgtACGGACCTAAAGTTCCCCCATAGGGGGGTACCTAAAGCCCAGCCAATCACCTAAAGTCAACCTTCGGTTGACCTTGAGGGTTCCCTAAGGGTTGGGGATGACCCTTGGGTTTGTCTTTGGGTGTTACCTTGAGTGTCTCTCTGTGTCCCTATCTGTTACAGTCTCCTAAAGTATCCTCCTAAAGTCACCTCCTAACGTCCATCCTAAAGCCAACACCTAAAGCCTACACCTAAAGACCCATCAAGTCAACGCCTATCTTAAAGTTTAAACAtaaagacctgtctcttatacacatct                                                                                                                                                                                                                                                                                     >  1:313782/1‑283 (MQ=255)`  
         `gtgtACGGACCTAAAGTTCCCCCATA‑GGGGGTACCTAAAGCCCAGCCAATCACCTAAAGTCAACCTTCGGTTGACCTTGAGGGTTCCCTAAGGGTTGGGGATGACCCTTGGGTTTGTCTTTGGGTGTTACCTTGAGTGTCTCTCTGTGTCCCTATCTGTTACAGTCTCCTAAAGTATCCTCCTAAAGTCACCTCCTAACGTCCATCCTAAAGCCAACACCTAAAGCCTACACCTAAAGACCCATCAAGTCAACGCCTATCTTAAAGTTTACACATaaagaccagacctaaagaccagacct                                                                                                                                                                                                                                                                                    >  1:9078/1‑301 (MQ=255)`  
         `gtgtACGGACCTAAAGTTCCCCCATA‑GGGGGTACCTAAAGCCCAGCCAATCACCTAAAGTCAACCTTCGGTTGACCTTGAGGGTTCCCTAAGGGTTGGGGATGACCCTTGGGTTTGTCTTTGGGTGTTACCTTGAGTGTCTCTCTGTGTCCCTATCTGTTACAGTCTCCTAAAGTATCCTCCTAAAGTCACCTCCTAACGTCCATCCTAAAGCCAACACCTAAAGCCTACACCTAAAGACCCATCAAGTCAACGCCTATCTTAAAGTTTAAACAtaaagaccagacctaaagacctgtctc                                                                                                                                                                                                                                                                                    >  1:234941/1‑295 (MQ=255)`  
         `gtgtACGGACCTAAAGTTCCCCCATA‑GGGGGTACCTAAAGCCCAGCCAATCACCTAAAGTCAACCTTCGGTTGACCTTGAGGGTTCCCTAAGGGTTGGGGATGACCCTTGGGTTTGTCTTTGGGTGTTACCTTGAGTGTCTCTCTGTGTCCCTATCTGTTACAGTCTCCTAAAGTATCCTCCTAAAGTCACCTCCTAACGTCCATCCTAAAGCCAACACCTAAAGCCTACACCTAAAGACCCATCAAGTCAACGCCTATCTT‑AAGTTTAAACATAaagaccagacctaaagaccagaccta                                                                                                                                                                                                                                                                                   >  1:291105/1‑301 (MQ=255)`  
         `gtgtACGGACCTAAAGTTCCCCCATA‑GGGGGTACCTAAAGCCCAGCCAATCACCTAAAGTCAACCTTCGGTTGACCTTGAGGGTTCCCTAAGGGTTGGGGATGACCCTTGGGTTTGTCTTTGGGTGTTACCTTGAGTGGCGCTCTGTGTCACTATATGTTACAGTCTCCTAAAGTATCCTCCTAAAGTCACCTCCTAACGTCCATCCTAAAGCCAACACCTAAAGCCTACAACTAAAGACCCATCAAGTCAACGCCTATCTTAAAGTTTAAACATaaagaccagacctaaagaccagacct                                                                                                                                                                                                                                                                                    >  1:137080/1‑301 (MQ=255)`  
         `gtgtACGGACCTAAAGTTCCCCCATA‑GGGGGTACCTAAAGCCCAGCCAATCACATAAAGTCAACCTTCGGTTGACCTTGAGGGTTCCCTAAGGGTTGGGGATGACCCTTGGGTTTGTCTTTGGGTGTTACCTTGAGTGTCTCTCTGTGTCCCTATCTGTTACAGTCTCCTAAAGTATCCTCCTAAAGTCACCTCCTAACGTCCATCCTAAAGCCAACACCTAAAGCCTACACCTAAAGACCCATCAAGTCAACGCCTATCTTAAAGTTTAAACATaaagaccagacctaaagaccagacct                                                                                                                                                                                                                                                                                    >  1:3493/1‑301 (MQ=255)`  
           `gtACGGACCTAAAGTTCCCCCATA‑GGGGGTACCTAAAGCCCAGCCAATCACCTAAAGTCAACCTTCGGTTGACCTTGAGGGTTCCCTAAGGGTTGGGGATGACCCTTGGGTTTGTCTTTGGGTGTTACCTTGAGTGTCTCTCTGTGTCCCTATCTGTTACAGTCTCCTAAAGTATCCTCCTAAAGTCACCTCCTAACGTCCATCCTAAAGCCAACACCTAAAGCCTACACCTAAAGACCCATCAAGTCAACGCCTATCTTCAAGTTTAAACAtaaagaccagacctaaagctgtcccttaa                                                                                                                                                                                                                                                                                  >  1:28762/1‑290 (MQ=255)`  
               `cagccTAAAGTTCCCCCATA‑GGGGGTACCTAAAGCCCAGCCAATCACCTAAAGTCAACCTTCGGTTGACCTTGAGGGTTCCCTAAGGGTTGGGGATGACCCTTGGGTTTGTCTTTGGGTGTTACCTTGAGTGTCTCTCTGAGTCCCTATCTGTTACAGTCTCCTAAAGTATCCTCCTAAAGTCACCTCCTAACGTCCATCCTAAAGCCAACACCTAAAGCCTACACCTAAAGACCCATCAAGTCAACGCCTATCTTAAAGTTTAAACATAAAGACcagacctaaagaccagacctaaagac                                                                                                                                                                                                                                                                              <  1:263160/298‑1 (MQ=255)`  
                `gACCTAAAGTTTCCCCATA‑GGGGGTACCTAAAGCCCAGCCAATCACCTAAAGTCAACCTTCGGTTGACCTTGAGGGTTCCCTAAGGGTTGGGGATGACCCTTGGGTTTGTCTTTGGGTGCTACCTTGAGTGTCTCTCTGTGTCCCTATCTGTTACAGTCTCCTAAAGTATCCTCCTAAAGTCACCTCCTAACGTCCATCCTAAAGCCAACACCTAAAGCCTACACCTAAAGACCCATCAAGTCAACGCCTATCTTAACGTTTAAACATAAAGACCAGACCTAAAGACCAGACCTAAAGaca                                                                                                                                                                                                                                                                             >  1:254707/1‑301 (MQ=255)`  
                `gACCTAAAGTTCCCCCATA‑GGGGGTATCTAAAGCCCAGCCAATCAGCTAAAGTCAACCTTCGGGTGACCTTGAGGGTTCCCTAAGGGTTGGGGATGACCCTTGGGTTTGTCTTTGGGTGTTACCTTGAGTGTCTCTCTGTGTCCCTATCTGTTACAGTCTCCTAAAGTATCCTCCTATAGTCACCTCCTAACTTCCATCCTAAATCCAACACCTAAAGCCTACACCTAAAGACCCATCATGTCAACGCCTATCTTACAGTTTTAACAtaaagacccaacctaaagctcagcacatgtccct                                                                                                                                                                                                                                                                             >  1:331926/1‑285 (MQ=21)`  
                `gACCTAAAGTTCCCCCATA‑GGGGGTACCTAAAGCCCAGCCAATCCCCTAAAGTCAACCTTCGGTTGACCTTGAGAGTTCCCTAAGAGTCGAGGATGACCCTTGGGTTTGTCTTTGGGTGTTACCTTGAGTGTCTCTCTGTGTTCCTATCTGTTACAGTTTCCTAAAGTATCCTCCTATAGTCACCTCCTAACGTCCATCCTAAATCCAACACCTAACGCCTACACCTAACGACCCATCAAGTCCACGCCTACCATAAAGCTTAAACATAAAGACCAGACTTCACGACCAGACCTAAAcaca                                                                                                                                                                                                                                                                             >  1:157905/1‑301 (MQ=21)`  
                `gACCTAAAGTTCCCCCATA‑GGGGGTACCTAAAGCCCAGCCAATCACCTAAAGTCAACCTTCGGTTGACCTTGAGGGTTCCCTAAGGGTTGGGGATGACCCTTGGGTTTGTCTTTGGGTGTTACCTTGAGTGTCTCTCTGTGTCCCTATCTGTTACAGTCTCCTAAAGTATCCTCCTAAAGTCACCTCCTAACGTCCATCCTAAAGCCAACACCTAAAGCCTACACCTAAAGACCCATCAAGTCAACGCCTATCTTAAAGTTTAAACAtaaagaccagacctaaagctgtctcttatacaca                                                                                                                                                                                                                                                                             >  1:317096/1‑285 (MQ=255)`  
                `gACCTAAAGTTCCCCCATA‑GGGGGTACCTAAAGCCCAGCCAATCACCTAAAGTCAACCTTCGGTTGACCTTGAGGGTTCCCTAAGGGTTGGGGATGACCCTTGGGTTTGTCTTTGGGTGTTACCTTGAGTGTCTCTCTGTGTCCCTATCTGTTACAGTCTCCTAAAGTATCCTCCTAAAGTCACCTCCTAACGTCCATCCTAAAGCCAACACCTAAAGCCTACACCTAAAGACCCATCAAGTCAACGCCTATCTTAAAGTTTAAACATAAAGAccagacctaaagacaagacctaaagaaa                                                                                                                                                                                                                                                                             >  1:180524/1‑299 (MQ=255)`  
                `gACCTAAAGTTCCCCCATA‑GGGGGTACCTAAAGCCCAGCCAATCACCTAAAGTCAACCTTCGGTTGACCTTGAGGGTTCCCTAAGGGTTGGGGATGACCCTTGGGTTTGTCTTTGGGTGTTACCTTGAGTGTCTCTCTGTGTCCCTATCTGTTACAGTCTCCTAAAGTATCCTCCTAAAGTCACCTCCTAACGTCCATCCTAAAGCCAACACCTAAAGCCTACACCTAAAGACCCATCAAGTCAACGCCTATCTTAAAGTTTAAACATAAAGACCAGACCTAAAGACCAGACCTAAAGaca                                                                                                                                                                                                                                                                             >  1:30730/1‑301 (MQ=255)`  
                `gACCTAAAGTTCCCCCATA‑GGGGGTACCTAAAGCACAGCCAATCACCTAAAGTCAACCTTCGGTTGACCTTGAGGGTTCCCTAAGGGTTGGGGATGACCCTTGGGTTTGTCTTTGGGTGTTACCTTGAGTGTCTCTCTGTGTCCCTATCTGTTACAGTCTCCTAAAGTATCCTCCTAAAGTCACCTCCTAACGTCCATCCTAAAGCCAACACCTAAAGCCTACACCTAAAGACCCATCAAGTCAACGCCTTTCTTAAAGTTTAAACAtaaagaccagacctaaagaccccttgttgttacc                                                                                                                                                                                                                                                                             >  1:145590/1‑288 (MQ=255)`  
                  `ccTAAAGTTCCCCCATA‑GGGGGTACCTAAAGCCCAGCCAATCACCTAAAGTCAACCTTCGGTTGACCTTGAGGGTTCCCTAAGGGTTGGGGATGACCCTTGGGTTTGTCTTTGGGTGTTACCTTGAGTGTCTCTCTGTGTCCCTATCTGTTACAGTCTCCTAAAGTATCCTCCTAAAGTCACCTCCTAACGTCCATCCTAAAGCCAACACCTAAAGCCTACACCTAAAGACCCATCAAGTCAACGCCTATCTTAAAGTTTAAACAtaaagaccagacctaaagacctgtctcttatacaca                                                                                                                                                                                                                                                                           >  1:114022/1‑286 (MQ=255)`  
                       `gtataagagacaggggggTACCTAAAGCCCAGCCAATCACCTAAAGTCAACCTTCGGTTGACCTTGAGGGTTCCCTAAGGGTTGGGGATGACCCTTGGGTTTGTCTTTGGGTGTTACCTTGAGTGTCTCTCTGTGTCCCTATCTGTTACAGTCTCCTAAAGTATCCTCCTAAAGTCACCTCCTAACGTCCATCCTAAAGCCAACACCTAAAGCCTACACCTAAAGACCCATCAAGTCAACGCCTATCTTAAAGTTTAAACATAAAGACCAGACCTAAAGACCAGACCTAAAGACACTACAt                                                                                                                                                                                                                                                                       <  1:97329/288‑1 (MQ=255)`  
                           `cccccATA‑GGGGGTACCTAAAGCCCAGCCAATCACCTAAAGTCAACCTTCGGTTGACCTTGAGGGTTCCCTAAGGGTTGGGGATGACCCTGGGGTTTGTCTTTGGGTGTTACCTTGAGTGTCTCTCTGTGTCCCTATCTGTTACAGTCTCCTAAAGTATCATCCTAAAGTCACCTCCTAACGTCCATCCTAAAGCCAACACCTAAAGCCTAAACCTAAAGACCCATCAAGTCAACGACTATCTTAAAGTTTAAACATAAAGACCAGACCTAAAGACCAGACCTAAAGACACTAAATAAaga                                                                                                                                                                                                                                                                  >  1:229567/1‑301 (MQ=255)`  
                            `ccccATA‑GGGGGTACCTAAAGCCCAGCCAATCACCTAATGTCAACCTTCGGTTGACCTTGAGGGTTCCCTAAGGGTTGGGGATGACCCTTGGGTTTGTCTTTGGGTGTTACCTTGAGTGTCTCTCTGTGTCCCTATCTGTTACAGTCTCCTAAAGTATCCTCCTAAAGTCACCTCCTAACGTCCATCCTAAAGCCAACACCTAAAGCCTACACCTAAAGACCCATCAAGTCAACGCCTATCTTAAAGTTTAAACATAAAGACCAGACCTAAAGACCAGACCTAAAGACACTACATAAagac                                                                                                                                                                                                                                                                 <  1:295885/301‑1 (MQ=255)`  
                            `caccATA‑GGGGGTACCTAAAGCCCAGCCAATCACCTAAAGTCAACCTTCGGTTGACCTTGAGGGTTCCCTAAGGGTTGGGGATGACCCTTGGGTTTGTCTTTGGGTGTTACCTTGAGTGTCTCTCTGTGTCCCTATCTGTTACAGTCTCCTAAAGTATCCTCCTAAAGTCACCTCCTAACGTCCATCCTAAAGCCAACACCTAAAGCCTACACCTAAAGACCCATCAAGTCAACGCCTATCTTAAAGTTTAAACATAAAGACCAGACCTAAAGACCAGACCTAAAGACACTACATAAagac                                                                                                                                                                                                                                                                 <  1:320020/299‑1 (MQ=255)`  
                             `cccATA‑GGGGGTACCTAAAGCCCAGCCAATCACCTAAAGTCAACCTTCGGTTGACCTTGAGGGTTCCCTAAGGGTTGGGGATGACCCTTGGGTTTGTCTTTGGGTATTACCTTGAGTGTCTCTCTGTGTCCCTATCTGTTACAGTCTCCTAAAGTATCCTCCTAAAGTCACCTCCTAACGTCCATCCTAAAGCCAACACCTAAAGCCTACACCTAAAGACCCATCAAGTCAACGCCTATCTTAAAGTTGAAACATAAAGACCAGACCTAAAGACCAAACCTAAAGGCACAACATAAagacc                                                                                                                                                                                                                                                                >  1:212273/1‑301 (MQ=255)`  
                              `ccATA‑GGGGGTACCTAAAGCCCAGCCAATCACCTAAAGTCAACCTTCGGTTGACCTTGAGGGTTCCCTAAGGGTTGGGGATGACCCTTGGGTTTGTCTTTGGGTGTTACCTTGAGTGTCTCTCTGTGTCCCTATCTGTTACAGTCTCCTAAAGTATCCTCCTAAAGTCACCTCCTAACGTCCATCCTAAAGCCAACACCTAAAGCCTACACCTAAAGACCCATCAAGTCAACGCCTATCTTAAAGTTTAAACATAAAGACCAGACCTAAAGACCAGACCTAAAGACACTACATAAagacca                                                                                                                                                                                                                                                               >  1:281733/1‑301 (MQ=255)`  
                                  `a‑GGGGGTACCTAAAGCCCAGCCAATCACCTAAAGTCAACCTTCGGTTGACCTTGAGGGTTCCCTAAGGGTTGGGGATGACCCTTGGGTTTGTCTTTGGGTGTTACCTTGAGTGTCTCTCTGTGTCCCTATCTGTTACAGTCTCCTAAAGTATCCTCCTAAAGTCACCTCCTAACGTCCATCCTAAAGCCAACACCTAAAGCCTACACCTAAAGACCCATCAAGTCAACGCCTATCTTAAAGTTTAAACATAAAGACCAGACCTAAAGACCAGACCTAAAGACACTACATAAagaccagacc                                                                                                                                                                                                                                                           <  1:222308/301‑1 (MQ=255)`  
                                     `ggggTACCTAAAGCCCAGCCAATCACCTAAAGTCAACCTTCGGTTGACCTTGAGGGTTCCCTAAGGGTTGGGGATGACCCTTGGGTTTGTCTTTGGGTGTTACCTTGAGTGTCTCTCTGTGTCCCTATCTGTTACAGTCTCCTAAAGTATCCTCCTAAAGTCACCTCCTAACGTCCATCCTAAAGCCAACACCTAAAGCCTACACCTAAAGACCCATCAAGTCAACGCCTATCTTAAAGTTTAAACATAAAGACCAGACCTAAAGACCAGACCTAAAGACACTACATAAAGACCAGACCTa                                                                                                                                                                                                                                                         >  1:131178/1‑301 (MQ=255)`  
                                           `ccTAAAGCCCAGCCAATCACCTAAAGTCAACCTTCGGTTGACCTTGAGGGTTCCCTAAGGGTTGGGGATGACCCTTGGGTTTGTCTTTGGGTGTTACCTTGAGTGTCTCTCTGTGTCCCTATCTGTTACAGTCTCCTAAATTATCCTCCTAAAGTCACCTCCTAACGTCCTTCCTAAAGCCAACACCTAAAGCCTACACCTAAAGACCCATCAAGTCAACGCCTATCTTAAAGTTTAAACATAAAGACCAGACCTAAAGACCAGACCTAAAGACCCTACATAAAGACCAGACCTCAAGAcc                                                                                                                                                                                                                                                   >  1:298546/1‑300 (MQ=255)`  
                                                 `gCCCAGCCAATCACCTAAAGGCAACCTTCGGTTGAACTTGAGGGTTCCCTAAGGGTTGGGGATGACCCTTGGGTTTGTCTTTGGGTGTTACCTTGAGTGTCTCTCTGTCTCCATATCTGTTACAGTCTCCTAAAGTATCCTTCTAAAGTAACCTCCTAACGTCCATCCTAAAGCCAACACCTAAAGCCTACAACTAAAGACACATCAAGTCAACGCCTATCTTAAAGTTTAAACATAAAGACCAGACCTAAAGACCAGACCTAAAGACACTACATAACGACCAGACCTAAAGACGCCttgt                                                                                                                                                                                                                                             >  1:257243/1‑301 (MQ=255)`  
                                                        `cAATCACCTAAAGTCAACCTTCGGTTGACCTTGAGGGTTCCCTAAGGGTTGGGGATGACCCTTGGGTTTGTCTTTGGGTGTTACCTTGAGTGTCTCTCTGTGTCCCTATCTGTTCCAGTCTCCTAAAGTATCCTCCTAAAGTCACCTCCTAACGTCCATCCTAAAGCCAACACCTAAAGCCTACACCTAAAGACCCATCAAGTCAACGCCTATCTTAAAGTTTAAACATAAAGACCAGACCTAAAGACCAGACCTAAAGACACTACATAAAGACCAGACCTAAAGACGCCTTGTTGTTAGc                                                                                                                                                                                                                                      <  1:43526/301‑1 (MQ=255)`  
                                                                 `aaaGTCAACCTTCGGTTGACCTTGAGGGTTCCCTAAGGGTTGGGGATGACCCTTGGGTTTGTCTTTGGGTGTTACCTTGAGTGTCTCTCTGTGTCCCTATCTGTTACAGTCTCCTAAAGTATCCTCCTAAAGTCACCTCCTAACGTCCATCCTAAAGCCAACACCTAAAGCCTACACCTAAAGACCCATCAAGTCAACGCCTATCTTAAAGTTTAAACATAAAGACCAGACCTAAAGACCAGACCTAAAGACACTACATAAAGACCAGACCTAAAGACGCCTTGTTGTTAGCCATAAAGTg                                                                                                                                                                                                                             >  1:231788/1‑301 (MQ=255)`  
                                                                       `aaCCTTCGGTTGACCTTGAGGGTTACATAAGGGTTGGGGATGACCCTTGGGTTTGTATTTGGGTGTTACCTTGAGTGTCTCGCTGTGTCCCTATCTGTTACAGTCTCCTAAAGTATCCTCCTAAAGTCACCTCCTAACGTCCATCCTAAAGCCAACACCTAAAGCCTACACCTAAAGACCCATCAAGTCAACGACTATCTTAAAGTTTAAACATAAAGACCAGACCTAAAGACCAGACATAAAGACACTACATAAAGACCAGACCTAAAGACGCCTTGTTGTTAGCCATAAAGTGATAAcc                                                                                                                                                                                                                       <  1:205371/301‑1 (MQ=255)`  
                                                                              `gtttGACCTGGAGGGTTCCCTAAGGGTTGGGGATGACTCTTGGGTTTGTCTTTGGGTGTTACCTTGAGTGTCTCTCTGTGTCCCTATCTGTTACAGTCTCCTAAAGTATCCTCCTAAAGTCTCCTCCTAACGTCCATCCTAAAGCCAACACCTAAAGCCTACACCTAAAGACCCAACAAGTCAACGCCTATCTTAAAGTTTAAACATAAAGACCAGACCTAAAGACCAGACCTAAAGACACTACATAAAGTCCAGACCTAAAGACGCCTTGTTGTTAGCCATAAAGTGATAACCTTTAATc                                                                                                                                                                                                                <  1:273045/299‑1 (MQ=255)`  
                                                                              `ggTTGGCCTTGAGGGTTCCCAAAGGGTTGGGGCTGACCCTTGGTTTTGTCTTTGGGTGTTACCTTGAGTGTCTCACTGTGTCCCGATCTGTTACAGTCTCCTAAAGTATCCTCCTAAAGTCACCTCCTAACGTCCATCCTAAAGCCAACCCCTAAAGCCTACACCTAAAGACCCATCAAGTCAACGCCTATCTTAAAGTTTAAACATAAAGACCAGACCTAAAGACCAGACCTAAAGACACTACATAAAGACCAGACCTAAAGACGCCTTGTTGTTAGCCATAAAGTGATAACCTTTAATc                                                                                                                                                                                                                <  1:249248/301‑1 (MQ=255)`  
                                                                              `ggTTGACCTTGAGGGTTCCCTAAGGGTTGGGGACGACCCTTGGGTTTTTCTTTGGGTGTTACCTTGAGTGTCTCTCTGTGTCCCTATCTGTTACAGTCTCCTAAAGTATCCTCCTAAAGTCACCTCCTAACGTCCATCCTAAAGCCAACACCTAAAGCCTACACCTAAAGACCCATCAAGTCAACGCCTATCTTAAAGTTTAAACATAAAGACCAGACCTAAAGACCAGACCTAAAGACACTACATAAAGACCAGACCTAAAGACGCCTTGTTGTTAGCCATAAAGTGATAACCTTTAATc                                                                                                                                                                                                                <  1:41161/301‑1 (MQ=255)`  
                                                                              `ggTTGACCTTGAGGGTTCCCGAAGGGTTGAGGATGACCCTTGGGTTTGTCTTTGGGTGTTACCTTGACTGTCTCTCTGTGTCCCGATCTGTTACAGGCTCCTACAGTATCCTCCTAAAGTCACCTCCTAACGTCCATCCTAAATCCAACACCTAAAGCCTACACCTAAAGACCCATCAAGTCAACGCCTATCTTATAGTTTAAACATAAAGACCAGACCTAAAGACCAGACCTAAAGACACTACATAAAGACCAGACCTAAAGACGCCTTGTTGTTAGCCATAAAGTGATAACCTTTAATc                                                                                                                                                                                                                <  1:214921/301‑1 (MQ=255)`  
                                                                                   `aCCTTTAGGGTTCCCTAAGGGTTGGGGATGACCCTTGGGTTTGTCTTTGGGTGTTACCTTGAGTGTCTCTCTGTGTCCCTATCTGTTACAGTCTCCTAAAGTATCCTCCTAAAGTCACCTCCTAACGTCCATCCTAAAGCAAACACCTAAAGCCTACACCTAAAGACCCATCAAGTCAACGCCTATCTTAAAGTTTAAACATAAAGACCAGACCTAAAGACCAAACCTAAAGACACTACATAAAGACCAGACATAAAGACGACATGTTGTTAGCCATAAAGTGATAACCTTTAATCATTGt                                                                                                                                                                                                           >  1:186309/1‑301 (MQ=255)`  
                                                                                    `ccTTGAGGGTTCCCTAAGGGTTGGGGATGACTCTTGGGTTTGTCTTTGGGTGTTACCTTGAGTGTCTCTCTGTGTCCCTATCTGTTACAG‑CTCCTAAAGTATCCTCCTAAAGTCACCTCCTAACGTCCATCCTAAAGCCAACACCTAAAGCCTACACCTAAAGACCCATCAAGTCAACGCCTATCTTAAAGTTTAAACATAAAGACCAGACCTAAAGACCAGACCTAAAGACACTACATAAAGACCAGACCTAAAGACGCCTTGTTGTTAGcctgtctcttatacacatctccgagcccac                                                                                                                                                                                                         >  1:301767/1‑273 (MQ=39)`  
                                                                                    `ccTTGAGGGTTCCCTAAGGGTTGGGGATGACCCTTGGGTTTGTCTTTGGGTGTTACCTTGAGTGTCTCTCTGTGTCCCTATCTGTTACAGTCTCCTAAAGTATTCTCCTAAAGTCACCTCCTAACGTCCATCCTAAAGCCAACACCTAAAGCCTACACCTAAAGACCCATCAAGTCAACGCCTATCTTAAAGTTTAATCCTAAAGACCAGACCTAAAGTCCAGACTTAAAGACACTACATAAAGACCAGACCTACACACCCCTGTTTGTTATCCCTAAAGTGCTAACCTTTACTcctgtct                                                                                                                                                                                                          >  1:174309/1‑295 (MQ=38)`  
                                                                                    `ccTTGAGGGTTCCCTAAGGGTTGGGGATGACCCTTGGGTTTGTCTTTGGGTGTTACCTTGAGTGTCTCTCTGTGTCCCTATCTGTTACAGTCTCCTAAAGTATCCTCCTAAAGTCACCTCCTAACGTCCATCCTAAAGCCAACATCTAAAGCCTACACCTAAAGACCCATCAAGTCAACGCCTATCTTAAAGTTTAAACATAAAGACCAGACCTAAAGACCAGACCTAAAGACACTACATAAAGACCAGACCTAAAGACGCCTTGTTGTTAGCCATAAAGTGATAACCTTTAATCATTGtt                                                                                                                                                                                                          >  1:328532/1‑300 (MQ=255)`  
                                                                                          `gggTTCCCTAAGGGTTGGGGATGACCCTTGGGTTTGTCTTTGGGTGTTACCTTGAGTGTCTCTCTGTGTCCCTATCTGTTACAGTCTCCTAAAGTATCCTCCTAAAGTCACCTCCTAACGTCCATCCTAAAGCCAACACCTAAAGCCTACACCTAAAGACCCATCAAGTCAACGCCTATCTTAAAGTTTAAACATAAAGACCAGACCTAAAGACCAGACCTAAAGACACTACATAAAGACCAGACCTAAAGACGCCTTGTTGTTAGCCATAAAGTGATAACCTTTAATCATTGTCTttatt                                                                                                                                                                                                    >  1:304606/1‑301 (MQ=255)`  
                                                                                          `gggTTCCCTAAGGGTTGGGGATGACCCTTGGGTTTGTCTTTGGGGTGTACCTTGAGTGTCTGTCTGTGTCCCTATCCGTTACAGTCTCCTAAAGTATCCTCCTAAAGGCACCTCCTAACGTCCATCCTAAAGCCAACACCTAAAGCCTACACCTAAAGACCCATCAAGTCAACGCCTATCTTAAAGTTTAAACATAAAGACCAGACCTAAAGACCAGACCTAAAGACACTACATAAAGACCAGACCTAAAGACGCCTTGTTGTTAGCCATAAAGTGATAACCTTTAATCATTGTCTttatt                                                                                                                                                                                                    <  1:337154/301‑1 (MQ=255)`  
                                                                                              `tCCCTAAGGGTTGGGGATGACCCTTGGGTTTGTCTTTGGGTGTTACCTTGAGTGTCTCTCTGTGTCCCTATCTGTTACAGTCTCCTAAAGTATCCTCCTAAAGTCACCTCCTAACGTCCATCCTAAAGCCAACACCTAAAGCCTACACCTAAAGACCCATCAAGTCAACGCCTATCTTAAAGTTTAAACATAAAGACCAGACCTAAAGACCAGACCTAAAGACACTACATAAAGACCAGACCTAAAGACGCCTTGTTGTTAGCCATAAAGTGATAACCTTTAATCATTGTCTTTATTAATa                                                                                                                                                                                                >  1:154386/1‑301 (MQ=255)`  
                                                                                               `cccTAAGGGTTGGGGATGACCCTTGGGTTTGTCTTTGGGTGTTACCTTGAGTGTCTCTCTGTGTCCCTCTCTGTTACAGTCTCCTAAAGTATCCTCCTCAAGTCACCTCCTAACGTCCATCCTAACGCCAACACCTAACGCCTACACCTCAATACCCATCAAGTCAACGCCTATCTTAAAGTTTAAACATACAGACCAGACCTAAAGACCACACCTAAAGACACTACATAAAGACCATACCTAAAGTCTCCTTGTTGTTATCCATAACGTTCTACCCTTTAATCCTTTTCTttatctgtct                                                                                                                                                                                               >  1:224574/1‑295 (MQ=38)`  
                                                                                               `cccTAAGGGTTGGGGATGACCCTTGGGTTTGTCTTTGGGTGTTACCTTGAGTGTCTCTCTGTGTCCCTATCTGTTACAGTCTCCTAAAGTATCCTCCTAAAGTCACCTCCTAACTTCCATCCTAAAGCCAACACCTAAAGCCTACACCTAAAGACCCATCAAGTCAACGCCTATCTTAAAGTTTAAACATAAAGACCAGACCTAAAGACCAGACCTAAAGACACTACATAAAGACCAGACCTAAAGACGCCTTGTTGTTAGCCATAAAGTGATAAACTTTAATAATTGTCTTTATTAATaa                                                                                                                                                                                               >  1:52962/1‑300 (MQ=255)`  
                                                                                               `cccTAAGGGTTGGGGATGACCCTTGGGTTTGTCTTTGGGTGTTACCTTGAGTGTCTCTCTGTGTCCCTATCTGTTACAGTCTCCTAAAGTATCCTCCTAAAGTCACCTCCTAACGTCCATCCTAAAGCCACCCCCTAACGCCTACACCTACAGACCCATCACGTCAACGCCTATCTTCAAGTTTAAACATAAAGACCAGACCTCAAGACCACACCTAAAGCCACTACATAAAGACCATACCTAAAGACGCCTTGTTGTTAGCCATCCAGTTATCACCTTTACTCATTGTCTTTATtactac                                                                                                                                                                                               >  1:340053/1‑301 (MQ=38)`  
                                                                                               `cccTAAGGGTTGGGGATGACCCTTGGGTTTGTCTTTGGGTGTTACCTTGAGTGTCTCTCTGTGTCCCTATCTGTTACAGTCTCCTAAAGTATCCTCCTAAAGTCACCTCCTAACGTCCATCCTAAAGCCAACACCTAAAGCCTACACCTAAAGACCCATCAAGTCAACGCCTATCTTAAAGTTTAAACATAAAGACCAGACCTAAAGACCAGACCTAAAGACACTACATAAAGACCAGACCTAAAGACGCCTTGTTGTTAGCCATAAAGTGATAACCTTTAATcctgtctcttatacaaat                                                                                                                                                                                               >  1:245823/1‑284 (MQ=255)`  
                                                                                               `cccTAAGGGTTGGGGATGACCCTTGGGTTTGTCTTTGGGTGTTACCTTGAGTGTCTCTCTGTGTCCCTATCTGTTACAGTCTCCTAAAGTATCCTCCTAAAGTCACCTCCTAACGTCCATCATAAAGCCAACACCTAAAGCCTACACCTAAAGACCCATCAAGTCAACGCCTATCTTAAAGTTTAAACATAAAGACCAGACCTAAAGACCAGACCTAAAGACACTACATAAAGACCAGACCTAAAGACGCCTTGTTGTTAGCCATAAAGTGATAACCTTTAATCATTGTCTTTATTAATaa                                                                                                                                                                                               >  1:124913/1‑300 (MQ=255)`  
                                                                                               `cccTAAGGGTTGGGGATAACCCTTGGGTTTGTCTTTTGGTGTTACCTTGAGTGTCTCTCTGTGTCCCTATCTCTTACAATCTCCTAAAGTATCCTCCTAAAGTCACCTCCTCACGTCCATCCTAAGGCCAACACCTAAAGCCTACACCTAAAGACCCATCAAGTCAACGCCTATCTTAAAGTTTAAACATAAAGACCATACCTAAAGACCAGACCTAAAGACACTACATAAAGACCAGACCTAAATACGCCTTGTTGTTAGCCATAAAGTGATAACCTTTAATCATTGTCTTTATTAATAc                                                                                                                                                                                               <  1:25249/301‑1 (MQ=255)`  
                                                                                                     `gggTTGGGGATGACCCTTGGGTTTGTCTTTGGGTGTTACCTTGAGTGTCTCTCTGTGTCCCTATCTGTTACAGTCTCATAAAGTATCCTCCTAAAGTCACCTCCTAACGTCCATCCTAAAGCCAACACCTAAAGCCTACACCTAAAGACCCATCAAGTCAACGCCTATCTTAAAGTTTAAACATAAAGACCTGACCTAAAGACCAAACCTAAAGACACTACATAAAGACCAGACCTAAAGACGCCTTGTTGTTAGCCATAAAGTGATAACCTTTAATCATTGTCTttattctgtctcttat                                                                                                                                                                                         >  1:345220/1‑290 (MQ=255)`  
                                                                                                      `ggTTGGGGATGACCTTTGGGTTTGTCTTTGGGTGTTACCTTGAGTGTCTCTCTGTGTCCCTATCTGTTACAGTCTCCTAAAGTATCCTCCTAATGTCACCTCCTAACGTCCATCCTAAAGCCAACACCTAAAGCCTACACCTAAAGACCCATCAAGTCAACGCCTATCTTAAAGTTTAAACATAAAGACCAGACCTAAAGACCAGACCTAAAGACACTACATAAAGACCAGACCTAAAGACGCCTTGTTGTTAGCCATAAAGTGATAACCTTTAATCATTGTCTTTATTAATACAACTcct                                                                                                                                                                                        >  1:16128/1‑299 (MQ=255)`  
                                                                                                       `gTTGGGGATGACCCTTGGGTTTGTCTTTGGGTGTTACCTTGAGTGTCTCTCTGTGTCCCTATCTGTTACAGTCTCCTAAAGTATCCTCCTAAAGTCACCTCCTAACGTCCATCCTAAAGCCAACACCTAAAGCCTACACCTAAAGACCCATCAAGTCAACGCCTATCTTAAAGTTTAAACATAAAGACCAGACCTAAAGACCAGACCTAAAGACACTACATAAAGACAAGACCTAAAGACCCCTTGTTGTTAGCCATAAAGGAATAACCTTAAACCATTGTCTTTATTAATACAACTCAcc                                                                                                                                                                                       >  1:18949/1‑300 (MQ=255)`  
                                                                                                            `ggATGACCCTTGGGTTTGTCTTTGGGTGTTACCTTGAGTGTCTCTCTGTGTCCCTATCTGTTACAGTCTCCTAAAGTATCCTCCTAAAGTCACCTCCTAACGTCCATCCTAAAGCCAACACCTAAAGCATACACCTAAAGACCCATCAAGTCAACGCCTATCTTAAAGTTTAAACATAAAGACCAGACCTAAAGACCAGACCTAAAGACACTACATAAAGACCAGACCTAAAGACGCCTTGTTGTTAGCCATAAAGTGATAAACTTTAATCATTGTCTTTATTAATACAACTCACGATAAg                                                                                                                                                                                  >  1:46655/1‑301 (MQ=255)`  
                                                                                                             `gATGACCCTTGGGTTTGTCTTTGGGTGTTACCTTGAGTGTCTCTCGGTGTCCCTATCTGTTACAGTCTCCTAAAGTATCCTCCTAAAGTCACCTCCTAACGTCCATCCTAAAGCCAACACCTAAAGCCTACACCTAAAGACCCATCAAGTCAACGCCTATCTTAAAGTTTAAACAGAAAGACCAGACCTAAAGACCAGACCTAAAGACACTACATAAAGACCAGACCTAAAGACGCCTTGTTGTTAGCCATAAAGTGATAACCTTTAATCATTGTCTTTATTAATACAACTCACTATAAgg                                                                                                                                                                                 <  1:103561/301‑1 (MQ=255)`  
                                                                                                             `gATGAACCTTGGGATTGTCGTGGGGTGTTACCGTGGGAGGTTCTCTTTGAACCTATCTGTGACAGTCTCCTCAAGTAACCTCCTAAAATCACCTCCTAGCGTCCATCCTAAAGCGAAGATCTAAAGCCAACACCTAAAGACCCATCAAGTAAACGCCTATCTTAAAGTTTAAACATAAAGACCAGACCTAAAGACCAGACCTAAAGGCACTACATAAAGACCAGACCTAAAGACGCCTTGTTGTTAGCCATAAAGTGATAACCTTTAATCATTGTATTTATTAATACAACTCACTATAAgg                                                                                                                                                                                 <  1:143572/301‑1 (MQ=255)`  
                                                                                                              `aTGACCCTTGGGTTTGTCTTTGGGTGTTACCTTGAGTGTCTCTCTGTGTCCCTATCTGTTACAGTCTCCTAAAGTATCCTCCTAAAGTCACCTCCTAACGTCCATCCTAAAGCCAACACCTAAAGCCTACACCTAAAGACCCATCAAGTCAACGCCTATCTTAAAGTTTAAACATAAAGACCAGACCTAAAGACCAGACCTAAAGACACTACATAAAGACCAGACCTAAAGACGCCTTGTTGTTAGCCATAAAGTGATAACCTTTAATCATTGTCTTTATTAATACAACTCACTATAAGga                                                                                                                                                                                <  1:79493/301‑1 (MQ=255)`  
                                                                                                              `aTGACCCTTGGGTTTGTCTTTGGGTGTTACCTTGAGTGTCTCTCTGTGTCCCTATCTGTTACAGTCTCCTAAAGTATCCTCCTAAAGTCACCTCCTAACGTCCATCCTAAAGCCAACACCTAAAGCCTACACCTAAAGACACATCAAGTCAACGCCTATCTTAAAGTTTAAACATAAAGACCAGAACTAAAGACCAGACCTAAAGACACTACATAAAGACCAGACCTAAAGACGCCTTGTTGTTAGCCATAAAGTGATAACCTTTAATCATTGTCTTTATTAATACAACTCACTATAAGga                                                                                                                                                                                <  1:140798/301‑1 (MQ=255)`  
                                                                                                                            `tGTCTTTGGGTGTTACCTTGAGTGTCTCTCTGTGTCCCTATCTGTTACAGTCTCCTAAAGTATCCTCCTAAAGTCACCTCCTAACGTCCATCCTTAAGCCAACACCTAAAGCCTACACCTAAAGACCCATCAAGTCAACGCCTATCTTAAAGTTTAAACATAAAGACCAGACCTAAAGACCAGACCTAAAGACACTACATAAAGACCAGACCTAAAGACGCCTTGTTGTTAGCCATAAAGTGATAACCTTTAATCATTGTCTTTATTAATACAACTCACTATAAGGAGAGACAACTTAAag                                                                                                                                                                  <  1:110903/301‑1 (MQ=255)`  
                                                                                                                             `gTCTTTGGGTGTTACCTTGAGTGTCTCTCTGTGTCCCTATCTGTTACAGTCTCCTAAAGTATCCTCCTAAAGTCACCTCCTAACGTCCATCCTAAAGCCAACACCTAAAGCCTACACCTAAAGACCCATCAAGTCAATGCCTATCTTAAAGTTTAAACATAAAGACCAGACCTAAAGACCAGACCTAAAGACACTACATAAAGACCAGACCTAAAGACGCCTTGTTGTTAGCCATAAAGTGATAACCTTTAATCATTGTCTTTATTAATACAACTCACTATAAggctgtctcttatacaca                                                                                                                                                                 >  1:147890/1‑285 (MQ=255)`  
                                                                                                                             `gTCTTTGGGTGTTACCTTGAGTGTCTCTCTGTGTCCCGATCTGTTACAGTCTCCTAAAGTATCCTCCTAAAGTCACCTCCTAACGTCCATCCTAAAGCCAACACCTAAAGCCTACACCTAAAGACCCATCAAGTCAACGCCTATCTTAAAGTTTAAAAATAAAGACCAGACCTAAAGACCAGACCTAAAGACACTACATAAAGACCAGACCTAAAGACGCATTGTTGTTAGCCATAAAGTGATAACCTTGAATCATTGTCTTTATTAATACAAATCACTATAAGGAGAGACAACTTAAaga                                                                                                                                                                 >  1:77625/1‑301 (MQ=255)`  
                                                                                                                                       `cgtgggctcggagatgtgtataagagacagcTGTTACAGTCTCCTAAAGTATCCTCCTAAAGTCACCTCCTAACGTCCATCCTAAAGCCAACACCTAAAGCCTACACCTAAAGACCCATCAAGTCAACGCCTATCTTAAAGTTTAAACATAAAGACCAGACCTAAAGACCAGACCTAAAGACACTACATAAAGACCAGACCTAAAGACGCCTTGTTGTTAGCCATAAAGTGATAACCTTTAATCATTGTCTTTATTAATACAACTCACTATAAGGAGAGACAACTTAAAGAGACTTAAAAg                                                                                                                                                       <  1:310757/271‑1 (MQ=255)`  
                                                                                                                                        `ttACCTTGAGTGTCTCTCTGTGTCCCTATCTGTTACAGTCTCCTAAAGTATCCTCCTAAAGTCACCTCCTAACGTCCATCCTAAAGCCAACACCTAAAGCCTACACCTAAAGACCCATCAAGTCAACGCCTATCTTAAAGTTTAAACATAAAGACCAGACCTAAAGACCAGACCTAAAGACACTACATAAAGACCAGACCTAAAGACGCCTTGTTGTTAGCCATAAAGTGATAACCTTTAATCATTGTCTTTATTAATACAACTCACTATAAGGAGAGACACCTTAAAGAGACTTAAAAGa                                                                                                                                                      >  1:275513/1‑301 (MQ=255)`  
                                                                                                                                        `gggctcggagatgtgtataagagacagaTCTGTTACAGTCTCCTAAAGTATCCTCCTAAAGTCACCTCCTAACGTCCATCCTAAAGCCAACACCTAAAGCCTACACCTAAAGACCCATCAAGTCAACGCCTATCTTAAAGTTTAAACATAAAGACCAGACCTAAAGACCAGACCTAAAGACACTACATAAAGACCAGACCTAAAGACGCCTTGTTGTTAGCCATAAAGTGATAACCTTTAATCATTGTCTTTATTAATACAACTCACTATAAGGAGAGACAACTTAAAGAGACTTAAAAGa                                                                                                                                                      >  1:183872/28‑301 (MQ=255)`  
                                                                                                                                         `tACCTTGAGTGTCTCTCTGTGTCCCTATCTGTTACAGTCTCCTAAAGTATCCTCCTAAAGTCACCTCCTAACGTCCATCCTAAAGCCAACACCTAAAGCCTACACCTAAAGACCCATCAAGTCAACGCCTATCTTAACGTTTAAACATAAAGACCAGACCTAAAGACCAGACCTAAAGACACTACATCAAGACCAGACCTAAAGCCGCCTTGTTGTTAGCCATAACGTGATCACCTTTAATCATTGTCTTTATTAATACAACTCACTATACGGAGAGACAACTTAAAGAGACTTACAAGAt                                                                                                                                                     >  1:65823/1‑301 (MQ=255)`  
                                                                                                                                          `aCCTTGAGTGTCTCTCTGTGTCCCTATCTGTTACAGTCTCCTAAAGTATCCTCCTAAAGTCACCTCCTAACGTCCATCCTAAAGCCAACACCTAAAGCCTACACCTAAAGACCCATCAAGTCAACGCCTATCTTAAAGTTTAAACATAAAGACCAGACCTAAAGACCAGACCTAAAGACACTACATAAAGACCAGACCTAAAGACGCCTTGTTGTTAGCCATAAAGTGATAACCTTTAATCATTGTCTTTATTAATACAACTCACTATAAGGAGAGACAACTTAAAGAAACTTAAAATAtt                                                                                                                                                    >  1:171374/1‑301 (MQ=255)`  
                                                                                                                                               `gAGTGTCTCTCTGTGTCCCTATCTGTTACAGTCTCCTAAAGTATCCTCCTAAAGTCACCTCCTAACGTCCATCCTAAAGCCAACACCTAAAGCCTACACCTAAAGACCCATCAAGTCAACGCCTATCTTAAAGTTTAAACATAAAGACCAGACCTAAAGACCAGACCTAAAGACACTACATAAAGACCAGACCTAAAGACGCCTTGTTGTTAGCCATAAAGTGATAACCTTTAATCATTGTCTTTATTAATACAACTCACTATAAGGAGAGACAACTTAAAGAGACTTAAAAGATTAAttt                                                                                                                                               >  1:6887/1‑301 (MQ=255)`  
                                                                                                                                                     `ctctctGTGTCCCTATCTGTTACAGTCTCCTAAAGTATCCTCCTAAAGTCACCTCCTAACGTCCATCCTAAAGCCAACACCTAAAGCCTACACCTAAAGACCCATCAAGTCAACGCCTATCTTAAAGTTTAAACATAAAGACCAGACCTAAAGACCAGACCTAAAGACACTACATAAAGACCAGACCTAAAGACGCCTTGTTGTTAGCCATAAAGTGATAACCTTTAATCATTGTCTTTATTAATACAACTCACTATAAGGAGAGACAACTTAAAGAGACTTAAAAGATTAATTTAAAAtt                                                                                                                                         >  1:168712/1‑301 (MQ=255)`  
                                                                                                                                                               `cccTATCTGTTACAGTCTCCTAAAGTATCCTCCTAAAGTCACCTCCTAACGTCCATCCTAAAGCCAACACCTAAAGCCTACACCTAAAGACCCATCAAGTCAACGCCTATCTTAAAGTTTAAACATAAAGACCAGACCTAAAGACCAGACCTAAAGACACTACATAAAGACCAGACGTAAAGACGCCTTGTTGTTAGCCATAAAGTGATAACCTTTAATCATTGTCTTTATTAATACAACTCACTATAAGGAGAGACAACTTAAAGAGCCTTAAAAGCTTAATTTAAAATTTATCaaaaaa                                                                                                                               >  1:100046/1‑300 (MQ=255)`  
                                                                                                                                                                   `aTCTGTTACAGTCTCCTAAAGTATCCTCCTAAAGTCACCTCCTAACGTCCATCCTAAAGCCAACACCTAAAGCCTACACCTAAAGACCCATCAAGTCAACGCCTATCTTAAAGTTTAAACATAAAGACCAGACCTAAAGACCAGACCTAAAGACACTACCTAAAGACAAGCCCTAAAGACGCCTTGTTGTTAGCCATAAAGTGATAACCTTTAATCATTGTCTTTATTAATACAACTAACTATAAGGAGAGAAAAATTACAGAGACTTAAAAGATTAATTTAAAATTTATCAAAAAGATTa                                                                                                                           >  1:134545/1‑301 (MQ=255)`  
                                                                                                                                                                      `tGTTACAGTCTCCTAAAGTATCCTCCTTAAGTCACCTCCTAACGTCCATCCTAAAGCCAACACCTAACGCCTACACCTAAAGACCCATCAAGTCAACGCCTAGCTTAAAGTTTAAACATAAAGACCAGACCTAAAGACCAGACCTAAAGACACTACATAAAGACCAGACCTAAAGACGCCTTGTTGTTAGCCATAAAGTGATAACCTTTAATCATTGTCTTTATTAATACAACTCACTATAAGGAGAGACAACTTAAAGAGACTTAAAAGATTAATTTAAAATTTATCAAAAAGAGTATTg                                                                                                                        <  1:27799/301‑1 (MQ=255)`  
                                                                                                                                                                              `tctcCTAAAGTATCCTCCTAAAGTCACCTCCTAACGTCCATCCTAAAGCCAACACCTAAAGCCTACACCTAAAGACCCATCAAGTCAACGCCTATCTTAAAGTTTAAACATAAAGACCAGACCTAAAGACCATACCTAAAGACACTACATAAAGACCAGACCTAAAGACGCCTTGTTGTTAGCCATAAAGTGATAACCTTTAATCATTGTCTTTATTAATACAACTCACTATAAGGAGAGACAACTTAAAGAGACTTAAAAGATTAATTTAAAATTTATCAAAAAGAGTATTGACTTAAAg                                                                                                                <  1:208200/301‑1 (MQ=255)`  
                                                                                                                                                                               `ctcCTAAAGTATCCTCCTAAAGTCACCTCCTAACGTCCATCCTAAAGCCAACACCTAAAGCCTACACCTAAAGACCCATCAAGTCAACGCCTATCTTAAAGTTTAAACATAAAGACCAGACCTAAAGACCAGACCTAAAGACACTACATAAAGACCAGACCTAAAGACGCCTTGTTGTTAGCCATAAAGTGATAACCTTTAATCATTGTCTTTATTAATACAACTCACTATAAGGAGAGACAACTTAAAGAGACTTAAAAGATTAATTTGAAATTTATCAAAAAGAGTATTGACTTAAAGt                                                                                                               >  1:11673/1‑301 (MQ=255)`  
                                                                                                                                                                               `ctcCTAAAGTATCCTCCTAAAGTCACCTCCTAACGTCCATCCTAAAGCCAACACCTAAAGCCTACACCTAAAGACCCATCAAGTCAACGCCTATCTTAAAGTTTAAACATAAAGACCAGACCTAAAGACCAGACCTAAAGACACTACATAAAGACCAGACCTAAAGACGCCTTGTTGTTAGCCATAAAGTGATAACCTTTAATCATTGTCTTTATTAATACAACTCACTATAAGGAGAGACAACTTAAAGAGACTTAAAAAATTAATTTAAAATTTATCAAAAAGAGTATTGACTaaaaaa                                                                                                               >  1:93856/1‑299 (MQ=255)`  
                                                                                                                                                                                    `aaaGTATCCTCCTAAAGTCACCTCCTAACGTCCATCCTAAAGCCAACACCTAAAGCCTACACCTAAAGACCCATCAAGTCAACGCCTATCTTAAAGTTTAAACATAAAGACCAGACCTAAAGACCAGACCTAAAGACACTACATAAAGACCAGACCTAAAGACGCCTTGTTGTTAGCCATAAAGTGATAACCTTTAATCATTGTCTTTATTAATACAACTCACTATAAGGAGAGACAACTTAAAGAGACTTAAAAGATTAATTTAAAATTTATCAAAAAGAGTATTGACTTAAAGTCTAAc                                                                                                          >  1:260160/1‑301 (MQ=255)`  
                                                                                                                                                                                    `aaaGTATCCTACTAAAGTCACCTCCTAACGTCCATCCTAAAGCCAACACCTAAAGCCTACACCTAAAGACCCATCAAGTCAACGCCTATCTTAAAGTTTAAACATAAAGACCAGACCTAAAGACCAGACCTAAAGACACTACATAAAGACCAGACCTAAAGACGCCTTGTTGTTAGCCATAAAGTGATAACCTTTAATCATTGTCTTTATTAATACAACTCACTATAAGGAGAGACAACTTAAAGAGACTTAAAAGATTAATTTAAAATTTATCAAAAAGAGTATTGACTTAAAGTCTAAc                                                                                                          >  1:227215/1‑301 (MQ=255)`  
                                                                                                                                                                                     `aaGTATCCTCCTAAAGTCACCTCCTAACGTCCATCCTAAAGCCAACACCTAAAGCCTACACCTAAAGACACATCAAGTCAACGCCTATCTTAAAGTTTAAACATAAAGACCAGACCTAAAGACCAGACCTAAAGACACTACATAAAGACCAGACCTAAAGACGCCTTGTTGTTAGCCATAAAGTGATAACCTTTAATCATTGTCTTTATTAATACAACTCACTATAAGGAGAGACAACTTAAAGAGACTTAAAAGATTAATTTAAAATTTATCAAAAAGAGTATTGACTTAAAGTCTAAcc                                                                                                         <  1:219333/301‑1 (MQ=255)`  
                                                                                                                                                                                     `aaGTATCCTCCTAAAGTCACCTACTAACGTCCATCCTAAAGCCAACACCTAAAGACTACACCTAAAGACCCATCAAGTCAACGCCTATCTTAAAGTTTAAACATAAAGACCAGACCTAAAGACCAGACCTAAAGACACTACATAAAGACCAGACCTAAAGACGCCTTGTTGTTAGCCATAAAGTGATAACCTTTAATCATTGTCTTTATTAATACAACTCACTATAAGGAGAGACAACTTAAAGAGACTTAAAAGATTAATTTAAAATTTATCAAAAAGAGTATTGACTTAAAGTCTAAcc                                                                                                         <  1:150524/301‑1 (MQ=255)`  
                                                                                                                                                                                          `tcctccTAAAGTCACCTCCTAACGTCCATCCTAAAGCCAACACCTAAAGCCTACACCTAAAGACCCATCAAGTCAACGCCTATCTTAAAGTTTAAACATAAAGACCAGACCTAAAGACCAGACCTAAAGACACTACATAAAGACCAGACCTAAAGACGCCTTGTTGTTAGCCATAAAGTGATAACCTTTAATCATTGTCTTTATTAATACAACTCACTATAAGGAGAGACAACTTAAAGAGACTTAAAAGATTAATTTAAAATTTATCAAAAAGAGTATTGACTTAAAGTCTAACCTATAg                                                                                                    <  1:195475/301‑1 (MQ=255)`  
                                                                                                                                                                                          `aagacagaaaGTCACCTCCTAACGTCCATCCTAAAGCCAACACCTAAAGCCTACACCTAAAGACCCCTCAAGTCAACGCCTATCTTAAAGTTTAAACATAAAGACCAGACCTAAAGACCAGACCTAAAGACACTACATAAAGACCAGACCTAAAGACGCCTTGTTGTTAGCCATAAAGTGATAACCTTTAATCATTGTCTTTATTAATACAACTCACTATAAGGAGAGACAACTTAAAGAGACTTAAAAGATTAATTTAAAATTTATCAAAAAGAGTATTGACTTAAAGTCTAACCTATAg                                                                                                    <  1:6209/294‑1 (MQ=255)`  
                                                                                                                                                                                            `ctcctAAAGTCACCTCCTAACGAACAACCTAAAGCCAACACCTAAAGCCTACCCCTAAAGACCCATCAAGTCAACGCCTATCTTAAAGTTTAAACATAAAGACCAGACCTAAAGACCAGACCTAAAGACACTACATAAAGACCAGCCCTAAAGACGCCTTGTTGTTAGCCATAAAGTGATAACCTTTAATCATTGTCTTTATTAATACAACTCACTATAAGGAGAGACAACTTAAAGAGACTTAAAAGATTAATTTAAAATTTATCAAAAAGAGTATTGACTTAAAGTCTAACCTATAGGa                                                                                                  <  1:127761/301‑1 (MQ=255)`  
                                                                                                                                                                                             `tcctAAAGTCACCTCCGAACGTCCATCCTAAAGCCAACACCTAAAGCCTACACCTAAAGACCCATCAAGTCAACGCCTATCTTAAAGTTTAAACATAAAGACCAGACCTAAAGACCAGACCTAAAGACACTACATAAAGACCAGACCTAAAGACGCCTTGTTGTTAGCCATAAAGTGATAACCTTTAATCATTGTCTTTATTAATACAACTCACTATAAGGAGAGACAACTTAAAGAGACTTAAAAGATTAATTTAAAATTTATCAAAAAGAGTATTGACTTAAAGTCTAACCTATAGGAt                                                                                                 <  1:193745/301‑1 (MQ=255)`  
                                                                                                                                                                                                         `ctcctAACGTCCATCCTAAAGCCAACACCTAAAGCCTCCACCTCAAGACCCATCAAGTCAACGCCTATCTTAAAGTTTAAACATAAAGACCAGACCTAAAGACCAGACCTAAAGACACTACATAAAGACCAGACCCCAAGCCGCCTTGTTGTTAGCCATAAAGTGATAACCTTTAATCATTGTCTTTATTAATACAACTCACTATACGGAGAGACAACTTAAAGAGACTTAAAAGATTAATTTAAAATTTATCAAAAAGAGTATTGACTTAAAGTCTAACCTATAGGATACTTCCAGcccc                                                                                     >  1:75256/1‑299 (MQ=255)`  
                                                                                                                                                                                                         `ctcctAACGTCCATCCTAAAGCCAACACCTAAAGCCTACACCTAAAGACCCATCAAGTCAACGCCTATCTTAAAGTTTAAACATAAAGACCAGACCTAAAGACCAGACCTAAAGACAGTACATAAAGACCAGACCTAAAGAAGCCGTGTTGTTAGCAATAAAGTGAGAACCTTTAATCATTGTCTTTATAAATACAACTCAATATAAGGAGAGACAACTTAAAGAGACTTAAAAGATTAATTTAAAATTTATCAAAAAGAGTATTGACTTAAAGTCAAACCTATAGGATACTTACAGCCAt                                                                                     >  1:1191/1‑301 (MQ=255)`  
                                                                                                                                                                                                                `cGTCCATCCTAAAGCCAAAACCTAAAGCCTACACCTAAAGACCCATCAAGTCAACGCCTATCTTAAAGTTTAAACATAAAGACCAGACCTAAAGACCAGACCTAAAGACACTACATAAAGACCAGACCTAAAGACGCCTTGTTGTTAGCCATAAAGTGATAACCTTTAATCATTGTCTTTATTAATACAACTCACTATAAGGAGAGACAACTTAAAGAGACTTAAAAGATTAATTTAAAATTTATCAAAAAGAGTATTGACTTAAAGTCTAACCTATAGGATACTTACAGCCATCGAGAgg                                                                              <  1:8637/301‑1 (MQ=255)`  
                                                                                                                                                                                                                       `ccTAAAGCCAACACCTAAAGCCTACACCTAAAGACCCATCAAGTCAACGCCTATCTTAAAGTTTAAACATAAAGACCAGACCTAAAGACCAGACCTAAAGACACTACATAAAGACCAGACCTAAAGACGCCTTGTTGTTAGCCATAAAGTGATAACCTTTAATCATTGTCTTTATTAATACAACTCACTATAAGGAGAGACAACTTAAAGAGACTTAAAAGATTAATTTAAAATTTATCAAAAAGAGTATTGACTTAAAGTCTAACCTATAGGATACTTACAGCCATCGAGAGGGACACgg                                                                       >  1:284640/1‑301 (MQ=255)`  
                                                                                                                                                                                                                       `ccTAAAGCCAACACCTAAAGCCTACACCTAAAGACCCATCAAGTCAACGCCTATCTTAAAGTTTAAACATAAAGACCAGACCTAAAGACCAGACCTAAAGACACTACATAAAGACCAGACCTAAAGACGCCTTGTTGTTAGCCATAAAGTGATAACCTTTAATCATTGTCTTTATTAATACAACTCACTATAAGGAGAGACAACTTAAAGAGAATTAAAAGATTAATTTAAAATTTATCAAAAAGAGTATTGACTTAAAGTCTAACCTATAGGATACTTACAGCCATCGAGAAGGACACgg                                                                       >  1:163939/1‑301 (MQ=255)`  
                                                                                                                                                                                                                                  `cacCTAAAGCCTACACCTAAAGACCCATCAAGTCAACGCCTATCTTAAAGTTTAAACATAAAGACCAGACCTAAAGACCAGACCTAAAGACACTACATAAAGACCAGACCTAAAGACGCCTTGTTGTTAGCCATAAAGTGATAACCTTTAATCATTGTCTTTATTAATACAACTCACTATAAGGAGAGACAACTTAAAGAGACTTAAAAGATTAATTTAAAATTTATCAAAAAGAGTATTGACTTAAAGTCTAACCTATAGGATACTTACAGCCATCTAGAGGGACACGGCCAATAGCCAt                                                            >  1:39037/1‑301 (MQ=255)`  
                                                                                                                                                                                                                                  `cacCTAAAGCCTACACCTAAAGACCCATCAAGTCAACGCCTATCTTAAAGTTTAAACATAAAGACCAGACCTAAAGACCAGACCTAAAGACACTACATAAAGACCAGACCTAAAGACGCCTTGTTGTTAGCCATAAAGTGATAACCTTTAATCATTGTCTTTATTAATACAACTCACTATAAGGAGAGACAACTTAAAGAGACTTAAAAGATTAATTTAAAATTTATCAAAAAGAGTATTGACTTAAAGTCTAACCTATAGGATACTTACAGCCATCGAGAGGGACACGGCGAATAGCCAt                                                            >  1:269695/1‑301 (MQ=255)`  
                                                                                                                                                                                                                                  `cacCTAAAGCCTACACCTAAAGACCCATCAAGTCAACGCCTATCTTAAAGTTTAAACATAAAGACCAGACCTAAAGACCAGACCTAAAGACACTACATAAAGACCAGACCTAAAGACGCCTTGTTGTTAGCCATAAAGTGATAACCTTTAATCATTGTCTTTATTAATACAACTCACTATAAGGAGAGACAACTTAAAGAGACTTAAAAGATTAATTTAAAATTTATCAAAAAGAGTATTGACTTAAAGTCTAACCTATAGGATACTTAAAGCCATCGAGAGGGACACGGCGAATAGCCaa                                                            >  1:55469/1‑300 (MQ=255)`  
                                                                                                                                                                                                                                    `ccTAAAGCCTACACCTAAAGACCCATCAAGTCAACGCCTATCTTAAAGTTTAAACATAAAGACCAGACCTAAAGACCAGACCTAAAGACACTACATAAAGACCAGACCTAAAGACGCCTTGTTGTTAGCCATAAAGTGATAACCTTTAATCATTGTCTTTATTAATACAACTCACTATAAGGAGAGACAACTTAAAGAGACTTAAAAGATTAATTTAAAATTTATCAAAAAGAGTATTGACTTAAAGTCTAACCTATAGGATACTTACAGCCATCGAGAGGGACACGGCGAATAGCCATcc                                                          <  1:242198/301‑1 (MQ=255)`  
                                                                                                                                                                                                                                          `gCCTACACCTAAAGACCCATCAAGTCAACGCCTATCTTAAAGTTTAAACATAAAGACCAGAACTAAAGACCAGACCTAAAGACACTACATAAAGACCAGACCTAAAGACGCCTTGTTGTTAGCCATAAAGTGATAACCTTTAATCATTGTCTTTATTAATACAACTCACTATAAGGAGAGACAACTTAAAGAGACTTAAAAGATTAATTTAAAATTTATCAAAAAGAGTATTGACTTAAAGTCTAACCTATAGGATACTTACAGCCATCGAGAGGGACACGGCGAATAGCCATCCCAATCg                                                    <  1:200720/301‑1 (MQ=255)`  
                                                                                                                                                                                                                                                 `ccTAAAGACCCATCAAGTCAACGCCTATCTTAAAGTTTAAACATAAAGACCAGACCTAAAGACCAGACCTAAAGACACTACATAAAGACCAGACCTAAAGACGCCTTGTTGTTAGCCATAAAGTGATAACCTTTAATCATTGTCTTTATTAATACAACTCACTATAAGGAGAGACAACTTAAAGAGACTTAAAAGATTAATTTAAAATTTATCAAAAAGAGTATTGACTTAAAGTCTAACCTATAGGATACTTACAGCCATCTAGAGGGACACGGCGAATAGCCATCCCAATCGACACCgg                                             >  1:293717/1‑301 (MQ=255)`  
                                                                                                                                                                                                                                                                 `gTCAACGCCTATCTTAAAGTTTAAACATAAAGACCAGACCTAAAGACCAGACCTAAAGACACTACATAAAGACCAGACCTAAAGACGCCTTGTTGTTAGCCATAAAGTGATAACCTTTAATCATTGTCTTTATTAATACAACTCACTATAAGGAGAGACAACTTAAAGAGACTTAAAAGATTAATTTAAAATTTATCAAAAAGAGTATTGACTTAAAGTCTAACCTATAGGATACTTACAGCCATCGAGAGGGACACGGCGAATAGCCATCCCAATCGACACCGGGGTCAACCGGATAAGt                             >  1:122327/1‑301 (MQ=255)`  
                                                                                                                                                                                                                                                                 `gTCAACGCCTATCTTAAAGTTTAAACATAAAGACCAGACCTAAAGACCAGACCTAAAGACACTACATAAAGACCAGACCTAAAGACGCCTTGTTGTTAGCCATAAAGTGATAACCTTTAATCATTGTCTTTATTAATACAACTCACTATAAGGAGAGACAACTTAAAGAGACTTAAAAGATTAATTTAAAATTTATCAAAAAGAGTATTGACTTAAAGTCTAACCTATAGGATACTTACAGCCATCGAGAGGGACACGGCGAATAGCCATCCCAATCGACACCGGGGTCAACCGGATAAGt                             >  1:10839/1‑301 (MQ=255)`  
                                                                                                                                                                                                                                                                   `taagagacagcTTAAAGTTTAAACATAAAGACCAGACCTAAAGACCAGACCTAAAGACACTACATAAAGACCAGACCTAAAGACGCCTTGTTGTTAGCCATAAAGTGATAACCTTTAATCATTGTCTTTATTAATACAACTCACTATAAGGAGAGACAACTTAAAGAGACTTAAAAGATTAATTTAAAATTTATCAAAAAGAGTATTGACTTAAAGTCTAACCTATAGGATACTTACAGCCATCGAGAGGGACACGGCGAATAGCCATCCCAATCGACACCGGGGTCAACCGGATAAGTAg                           <  1:49461/291‑1 (MQ=255)`  
                                                                                                                                                                                                                                                                     `aCGCCTATCTGAAAGTTTAAACATAAAGACCAGACCTAAAGAACAGACCTAAAGACACTACATAAATACCAGACCTAAAGACGCCTTGTTGTGAGCCATAAAGTGATAACCTTTAATCATTGTCTTTATTAATACAACTCACTATAAGGAGAGACAACTTAAAGAGACTTAAAAGATTAATTTAAAATTTATCAAAAAGAGTATTGACTTAAAGTCTAACCTATAGGATACTTACAGCCATCGAGAGGGACACGGCGAATAGCCATCCCAATCGACACCGGGGTCAACCGGATAAGTAGAc                         <  1:238753/301‑1 (MQ=255)`  
                                                                                                                                                                                                                                                                     `aCGACTAACGTAAAGGTTAAACAGAAAAGACAGACCTAAAGACCAGAACTAAAGACACGACATAAAGACATGACATAAAGACGACTTGTTGTTAGCAATAAAGTGATAACCTTTAATCATTGTCTTTATTAATACAACTCACTATAAAGAGAGACAACTTAAAGAGACTTAAAAGATTAATTTAAAATTTATCAAAAAGAGTATTGACTTAAAGTCTAACCTATAGGATACTTACAGCCATCGAGAGGGACACGGCGAATAGCCATCCCAATCGACACCGGGGTCAACCGGATAAGTAGAc                         <  1:82881/301‑1 (MQ=255)`  
                                                                                                                                                                                                                                                                           `aTCTTAAAGTTTAAACATAAAGACCAGACCTAAAGACCAGACCTAAAGACACTCCATAAAGACCCGACCTAAAGACGCCTTGTTGTTAGCCATAAAGTGATAACCTTTAATCATTGTCTTTATTTATACAACTCACTATAAGGAGAGACAACTTAAAGAGACCTAAAAGTTTAATTTAACCTTTATCAACAAGAGTATTGACTTAAAGTCTAACCTATAGGATACTTACAGCCATCGAGAGCGACACGGCGAATATCCATCCCAATCGACACCGCGGTCAACCGGCTAAGTAGACAGCATg                   >  1:177175/1‑301 (MQ=255)`  
                                                                                                                                                                                                                                                                           `aTCTTAAAGTTTAAACATAAAGACCAGACCTAAAGACCAGACCTAAAGACACTACATAAAGACCAGGCCTAAAGACGCCTTGTTGTTAGCCATAAAGTGATAACCTTTAATCATTGTCTTTATTAATACAACTCACTATAAGGAGAGACAACTTAAAGAGACTTAAAAGATTAATTTAAAATTTATCAAAAAGAGTATTGACTTAAAGTCTAACCTATAGGATACTTACAGCCATCGAGAGGGACACGGCGAATAGCCATCCCAATCGACACCGGGGTCAACCGGATAAGTAGACAGCCTg                   >  1:5947/1‑301 (MQ=255)`  
                                                                                                                                                                                                                                                                               `tAAAGTTTAAACATAAAGACCAGACCTAAAGACCAGACCTAAAGACACTACATAAAGACCAGACCTAAAGACGCCTTGTTGTTAGCCATAAAGTGATAACCTTTAATCATTGTCTTTATTAATACAACTCACTATAAGGAGAGACAACTTAAAGAGACTTAAAAGATTAATTTAAAATTTATCAAAAAGAGTATTGACTTAAAGTCTAACCTATAGGATACTTACAGCCATCGAGAGGGACACGGCGAATAGCCATCCCAATCGACACCGGGGTCAACCGGATAAGTAGACAGCCTGATaa               <  1:266195/301‑1 (MQ=255)`  
                                                                                                                                                                                                                                                                               `tAAAGTTTAAACATAAAGAACAGACCTAAAGACCAGACCTAAAGACACTACATAAAGACCAGACCTAAAGACGCCTTGTTGTTAGCCATAAAGTGATAACCTTTAATCATTGTCTTTATTAATACAACTCACTATAAGGAGAGACAACTTAAAGAGACTTAAAAGATTAATTTAAAATTTATCAAAAAGAGTATTGACTTAAAGTCTAACCTATAGGATACTTACAGCCATCGAGAGGGACACGGCGAATAGTCATCCCAATCGACACCGGGGTCAACCGGATAAGTAGACAGCCTGATaa               <  1:197594/301‑1 (MQ=255)`  
                                                                                                                                                                                                                                                                               `tAAAGGTTAAACATAAAGACCAGACCTAAAGACCAGACCTAAAGACACTACATAAAGACCAGACCTAAAGACGCCTTGTTGTTAGCCATAAAGTGATAACCTTGAATCATTGTCTTTATTAATACAACTCACTATAAGGAGAGACAACTTAAAGAGACTTAAAAGATTAATTTAAAATTTATCAAAAAGAGTATTGACTTAAAGTCTAACCTATAGGATACTTACAGCCATCGAGAGGGACACGGCGAATAGCCATCCCAATCGACACCGGGGTCAACCGGATAAGTAGACAGCCTGATaa               <  1:323679/301‑1 (MQ=255)`  

                                                                                                                                                                                                                                                                                           `|`                                                                                                                                                                                                                                                                                                               
`‑‑TCTCACAGTGTACGGACCTAAAGTTCCCCCATA‑GGGGGTACCTAAAGCCCAGCCAATCACCTAAAGTCAACCTTCGGTTGACCTTGAGGGTTCCCTAAGGGTTGGGGATGACCCTTGGGTTTGTCTTTGGGTGTTACCTTGAGTGTCTCTCTGTGTCCCTATCTGTTACAGTCTCCTAAAGTATCCTCCTAAAGTCACCTCCTAACGTCCATCCTAAAGCCAACACCTAAAGCCTACACCTAAAGACCCATCAAGTCAACGCCTATCTTAAAGTTTAAACATAAAGACCAGACCTAAAGACCAGACCTAAAGACACTACATAAAGACCAGACCTAAAGACGCCTTGTTGTTAGCCATAAAGTGATAACCTTTAATCATTGTCTTTATTAATACAACTCACTATAAGGAGAGACAACTTAAAGAGACTTAAAAGATTAATTTAAAATTTATCAAAAAGAGTATTGACTTAAAGTCTAACCTATAGGATACTTACAGCCATCGAGAGGGACACGGCGAATAGCCATCCCAATCGACACCGGGGTCAACCGGATAAGTAGACAGCCTGATAAGTCGCACGAAAAA  >  NC_001604.1/1‑582`

  

Alignment Legend

Aligned base mismatch/match (shaded by quality score): `ATCG/ATCG` `< 3 ≤` `ATCG/ATCG` `< 11 ≤` `ATCG/ATCG` `< 22 ≤` `ATCG/ATCG` `< 34 ≤` `ATCG/ATCG` `< 38 ≤` `ATCG/ATCG`

Unaligned base: `atcg`    Masked matching base: `atcg`    Alignment gap: `‑`    Deleted base: `‑`

Reads not counted as support for junction

read\_name Not counted due to insufficient overlap past the breakpoint.

read\_name Not counted due to not crossing MOB target site duplication.
